# Forest malaria in Cambodia: the occupational and spatial clustering of *Plasmodium vivax* and *Plasmodium falciparum* infection risk in a cross-sectional survey in Mondulkiri province, Cambodia

**DOI:** 10.1186/s12936-020-03482-4

**Published:** 2020-11-19

**Authors:** Mirco Sandfort, Amélie Vantaux, Saorin Kim, Thomas Obadia, Anaïs Pepey, Soazic Gardais, Nimol Khim, Dysoley Lek, Michael White, Leanne J. Robinson, Benoit Witkowski, Ivo Mueller

**Affiliations:** 1 Malaria: Parasites and Hosts Unit, Institut Pasteur, Paris, France; 2 Sorbonne Université, Collège doctoral, Paris, France; 3 Malaria Molecular Epidemiology Unit, Institut Pasteur du Cambodge, Phnom Penh, Cambodia; 4 Hub de Bioinformatique et Biostatistique, Département Biologie Computationnelle, Institut Pasteur, USR 3756 CNRS, Paris, France; 5 National Centre for Parasitology, Entomology, and Malaria Control, Phnom Penh, Cambodia; 6 School of Public Health, National Institute of Public Health, Phnom Penh, Cambodia; 7 Population Health & Immunity, Walter and Eliza Hall Institute, Melbourne, Australia; 8 University of Melbourne, Melbourne, Australia; 9 Burnet Institute, Melbourne, Australia

**Keywords:** Forest, Occupational risk, Spatial, Vivax, Hotspots, Cambodia, Greater Mekong Subregion

## Abstract

**Background:**

After a marked reduction in malaria burden in Cambodia over the last decades, case numbers increased again in 2017–2018. In light of the national goal of malaria elimination by 2025, remaining pockets of high risk need to be well defined and strategies well-tailored to identify and target the persisting burden cost-effectively. This study presents species-specific prevalence estimates and risk stratification for a remote area in Cambodia.

**Methods:**

A cross-sectional survey was conducted in 17 villages in the high-incidence province Mondulkiri in the dry season (December 2017 to April 2018). 4200 randomly selected participants (2–80 years old) were tested for *Plasmodium* infection by PCR. Risk of infection was associated with questionnaire-derived covariates and spatially stratified based on household GPS coordinates.

**Results:**

The prevalence of PCR-detectable *Plasmodium* infection was 8.3% (349/4200) and was more than twice as high for *Plasmodium vivax* (6.4%, 268) than for *Plasmodium falciparum* (3.0%, 125, *p* < 0.001). 97.8% (262/268) of *P. vivax* and 92.8% (116/125, *p* < 0.05) of *P. falciparum* infections were neither accompanied by symptoms at the time of the interview nor detected by microscopy or RDT. Recent travels to forest sites (aOR 2.17, *p* < 0.01) and forest work (aOR 2.88, *p* < 0.001) were particularly strong risk factors and risk profiles for both species were similar. Large village-level differences in prevalence of *Plasmodium* infection were observed, ranging from 0.6% outside the forest to 40.4% inside. Residing in villages at the forest fringe or inside the forest compared to outside was associated with risk of infection (aOR 2.14 and 12.47, *p* < 0.001). Villages inside the forest formed spatial hotspots of infection despite adjustment for the other risk factors.

**Conclusions:**

Persisting pockets of high malaria risk were detected in forested areas and in sub-populations engaging in forest-related activities. High levels of asymptomatic infections suggest the need of better case detection plans and the predominance of *P. vivax* the implementation of radical cure. In villages inside the forest, within-village exposure was indicated in addition to risk due to forest activities. Village-level stratification of targeted interventions based on forest proximity could render the elimination efforts more cost-effective and successful.

## Background

In the last two decades, Cambodia has seen a marked decrease in numbers of reported malaria cases from approximately 140,000 cases in 1999 to 60,000 in 2018 [1]. Likewise, mortality by malaria steadily reduced, with zero deaths reported for the first time in 2018. Similarly remarkable reductions have been observed in the other countries of the Greater Mekong Subregion [2]. Since 2014, these countries have even pursued the goal of elimination of all malaria by 2030 [3].

This ambitious aim however faces several obstacles. After an all-time low of approximately 20,000 reported cases in 2016, numbers have increased again in 2017–2018 [1, 2]. While the annual case numbers increased for both *Plasmodium falciparum* and *Plasmodium vivax* in 2017, they declined for *P. falciparum* in 2018 but increased further for *P. vivax* [1, 3]. Consequently, *P. vivax* now accounts for almost three-quarters of all malaria cases in Cambodia [2, 4].

The national malaria control programme (NMCP) is based on mass distribution campaigns of long-lasting insecticidal nets (LLIN), diagnosis by light microscopy (LM) and rapid diagnostic tests (RDT) in the public health sector, RDT-based testing by village/mobile malaria workers (VMW/MMW), and treatment of blood stage infections with artemisinin-based combination therapy (ACT) [5]. The spread of multi-drug resistant *P. falciparum* malaria in Western Cambodia [6] might explain the resurgence of *P. falciparum* cases in 2017 and the change of first-line treatment from dihydroartemisinin-piperaquine to artesunate-mefloquine their subsequent decline in 2018. For *P. vivax*, such resistance is not reported. However, this species has dormant liver-stages called hypnozoites that escape acute blood stage treatment and cause relapsing infections. Therefore, although LLINs do reduce *P. vivax* transmission and ACT is effective against blood stage *P. vivax* infection, neither intervention targets the hypnozoite reservoir. Hence, there is a less marked effect of these interventions on *P. vivax* than *P. falciparum*.

The only licensed drug with hypnozoiticidal effect is primaquine, typically administered at 0.25 mg/kg daily over 14 days in Cambodia. The roll-out of *P. vivax* radical cure is part of the national treatment guidelines but has not yet been implemented because of concerns over the drug’s potential haemolytic effect in patients with G6PD deficiency [7, 8]. In summary, the control and elimination efforts of the NMCP are successfully targeting clinical *P. falciparum* malaria (understandably in the context of drug-resistant *P. falciparum* [6] in the country) but to a lesser extent to *P. vivax*.

Another obstacle to elimination is the high prevalence of asymptomatic infections [9–12]. Neither health facilities nor VMWs reach these asymptomatic carriers as they do not seek diagnosis or treatment. Although of lower infectivity [13, 14], they remain however a potential source of onward transmission.

Alongside the sustained reduction in countrywide incidence, malaria has also become increasingly fragmented, affecting fewer, usually remote provinces. The highest incidence of malaria in Cambodia and across the Greater Mekong Subregion is now particularly found in the North-Eastern provinces such as Ratanakiri and Mondulkiri [1, 3]. These settings are characterized by a high proportion of ethnic minorities and agricultural or wood-logging activities as the main income sources. Exposure to *Anopheline* mosquitoes and *Plasmodium* in Cambodia is understood to primarily occur in the forest rather than peri-domestically [15–17].

Together with the Western province Pursat, the highest incidence per province in 2018 was reported for Mondulkiri [1]. However, previous studies on population-level burden and risk of malaria in Cambodia focused on the country’s West and North [12, 16–21] or the Eastern province of Ratanakiri [10, 11, 15, 20, 22, 23]. Here, using a cross-sectional survey conducted in a rural area of Mondulkiri in the dry season in 2018, estimates of the prevalence of *Plasmodium* infection are shown and key risk factors identified.

## Methods

### Study area and census

Seventeen villages were selected in the Kaev Seima district, Mondulkiri province, in the Kingdom of Cambodia. The rainy season in Cambodia normally runs from June to October with the high transmission period from June through December [1, 4, 24]. Approx. two-thirds of the population in Mondulkiri is comprised of national ethnic minorities, with the Phnong ethnic group comprising the largest proportion [25, 26]. In November–December 2017, a census was conducted by visiting each household in the 17 villages and collecting basic demographics of household participants such as age and gender. A person’s household was defined as location of main residence in the village according to adult members of the household or the village head. GPS coordinates were collected using Garmin® GPSMAP® 64s devices. When no adult household member could be found, demographic information was obtained from the village head’s registry book.

### Cross-sectional survey

Based on the census, a random selection of households was drawn oversampling small villages to ensure sufficient coverage (Additional file 1: Table ST1). Selected households were visited from mid-December 2017 until mid-April 2018 and all household members aged 2–80 years who had resided in the study area for at least 3 months were invited to participate in the survey. Upon informed consent, a questionnaire on household variables was administered to the head of household or another adult household member by trained interviewers. A questionnaire on individual-level variables was administered to each consenting household member. Children were interviewed assisted by a parent (or rarely another caregiver in the absence of the parents). Data were collected on tablets and run through automated data quality checks within days after the interview. In case of missing data or discrepancies, the field team was informed for immediate resolution if possible. Finger-prick blood samples were collected as thick and thin film slides and in K^+^EDTA-microtainers. Participants were screened for symptoms, *i.e.* feeling sick or feverish on the day of interview, having felt feverish over the preceding two days, or having an axillary body temperature of at least 37.5 °C. Upon any indication, the participant was administered a standard malaria RDT (Malaria Ag P.f/P.v, Standard Diagnostics Inc., South Korea) and referred to a local health care provider for treatment if positive.

### Detection of infection

The microtainer blood samples collected at the interview site were stored in 4 °C ice boxes. At a field laboratory, they were separated into plasma and cell pellet and frozen at −20 °C. Following transport to the main laboratory at Institut Pasteur of Cambodia in Phnom Penh, cell pellets were stored at −20 °C and plasma at −80 °C. Infections with any of the four human *Plasmodium* parasites were determined by real-time PCR [27]. In case of a positive genus-specific result, qPCR specific for *P. falciparum*, *P. vivax*, *Plasmodium malariae*, and *Plasmodium ovale* followed. All positive and a random selection of 10% negative samples were assessed by independent double LM readings of asexual and sexual stages and parasite densities calculated. No discrepancy in parasite densities of above 30% occurred. Parasites were counted per approximately 500 leukocytes and densities were inferred assuming 8000 leukocytes per microlitre blood.

### Descriptive analyses

Villages were classified based on the forest cover in a 750 m radius around the households, computed from the land cover analysis in [28] (Additional file 1: Figure S1). Villages with ≥ 50% of households with ≥ 10% forest cover in their vicinity were considered “inside the forest”, with ≥ 30% of households with ≥ 5% forest cover as “at the forest fringe”, or “outside the forest” otherwise. Because of very low sample sizes, the two small, neighbouring villages Beng (11 individuals) and Gaty (95 individuals) were analysed as one. Population prevalence was estimated based on post-sampling weights assigned to each participant according to their representation by village, gender, and 10-year age bins compared to the census population (raw numbers of positive survey samples accompany the estimates in brackets). Categorical covariates were compared using the chi-squared test or the Fisher’s exact test when low strata sizes required it.

### Risk factor analyses

The association of covariates with infection by *P. vivax*, *P. falciparum*, or all four species was assessed by mixed-effects logistic regression with random intercepts per household and village. Those covariates that were statistically significantly associated with *Plasmodium* infection at two-tailed α = 5% in univariate regressions were included in the multivariate model. The villages’ proximity to the forest, work-unrelated overnight travels (incl. to forest sites), and work in the deep forest were considered part of the multivariate model a priori in order to assess the association of forest exposure with risk of infection*.* Having slept outdoors or under a bed net last night were kept as part of the model based on causal reasoning or prior knowledge due to their association with infection risk in previous publications, e.g. [11]. Gender and age were retained in the model as proxies for risk-related behaviour and thus potential confounders. The other covariates comprised potential proxies for socioeconomic status or further exposure variables and their subset significantly associated with infection risk was assessed by backwards variable selection. Akaike Information Criterion (AIC) was used to assess model fit. If collinearities and interactions were identified, covariates were retained comparing fit of the respective models by AIC. Statistical significance in the multivariate regressions was calculated per covariate by likelihood ratio tests. Spatial hotspots were identified by a purely spatial scan statistic via a discrete Poisson model with maximally a third of the population in a scanning window, adjusted for all covariates of the final multivariate model except for the villages’ forest proximity [29].

### Software

All questionnaires were applied on tablets using the REDCap (Research Electronic Data Capture) software hosted at Institut Pasteur in Paris [30, 31]. Data quality control as well as all descriptive and analytical statistics were performed in R 3.6.3 [32]. The SaTScan™ software version 9.6 was used for the spatial scan statistic [33].

## Results

### Census, survey representativeness and survey population

**Fig. 1:**
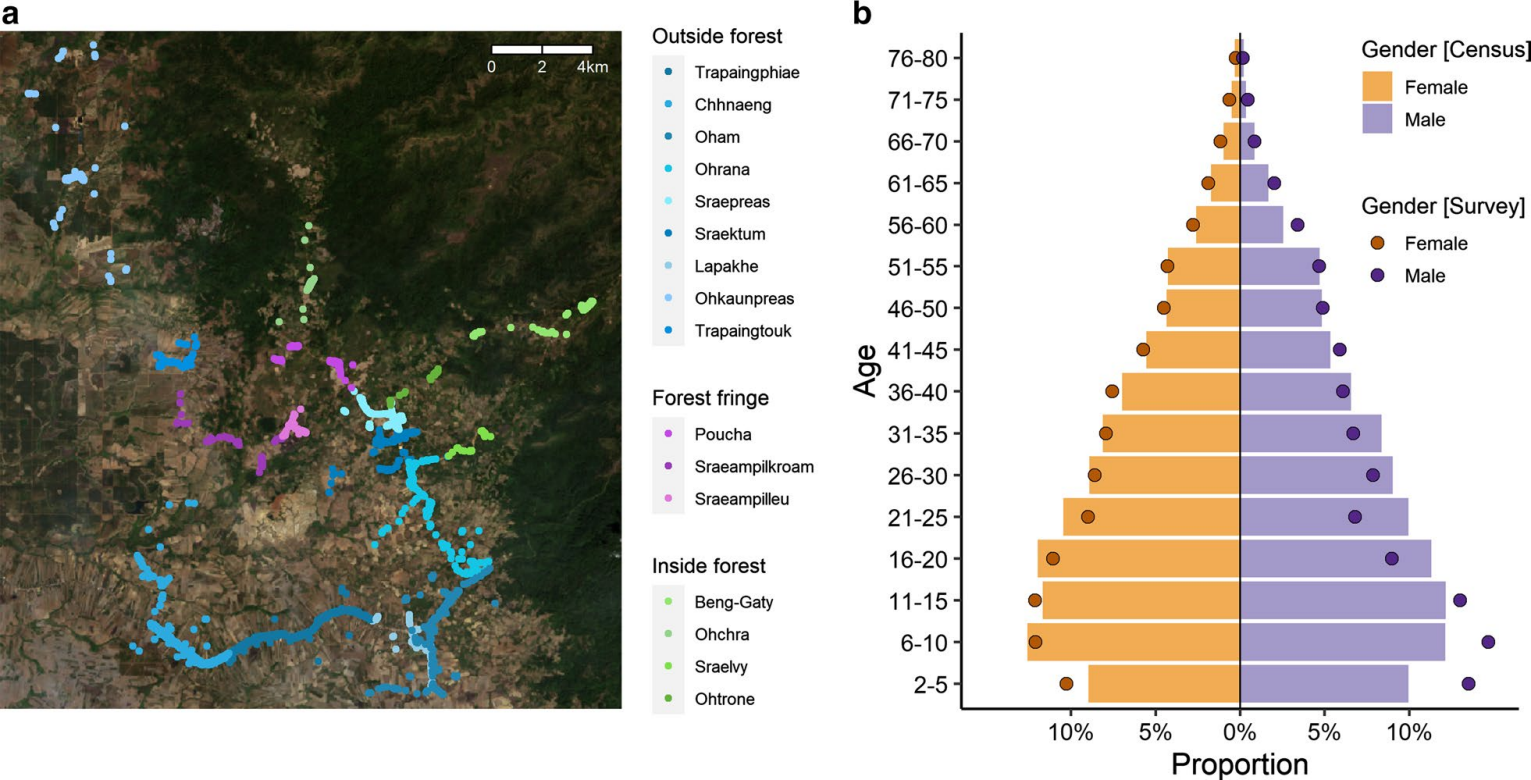
Geographic and demographic description of survey population. **a** Map of survey household locations, coloured by village in shades of blue, purple, or green if village is in category “outside forest”, “forest fringe”, or “inside forest”, respectively. Background Landsat-8 image courtesy of the U.S. Geological Survey. **b** Representativeness of age distribution in women and men by overlaying distribution in census (bars) by distribution in survey (points)

From among the 10,053 individuals in 2351 households identified in the census, the survey recruited 4200 participants from 1147 households and oversampled smaller villages on average (Additional file 1: Table ST1). A map of the survey households and the categorization of villages by proximity to the forest is shown in Fig. 1a. Mean age was 26 years in both the census and the survey and with 51% (5135/10,053) and 53% (2231/4200) women, respectively, women were slightly oversampled in the survey. In men, ages of 16 to 40 years were mildly underrepresented (Fig. 1b). The main income source for the survey households was by large majority farming (89.8%, 1030/1147). In terms of mobility, three-quarters (75.9%, 3188/4200) of the survey participants reported work-unrelated trips to forest or field sites in the last month (8.5%, 358/4200) or any work trip in the last two months (75.8%, 3184/4200). By vast majority, these trips were short and frequent: In only 4.4% (140/3188) of the instances, stays for longer than a week were reported and among those who reported any work trip, 89.4% (2847/3184) went for work at least once a week.

### *Plasmodium vivax* predominated over other human malaria parasites

**Fig. 2:**
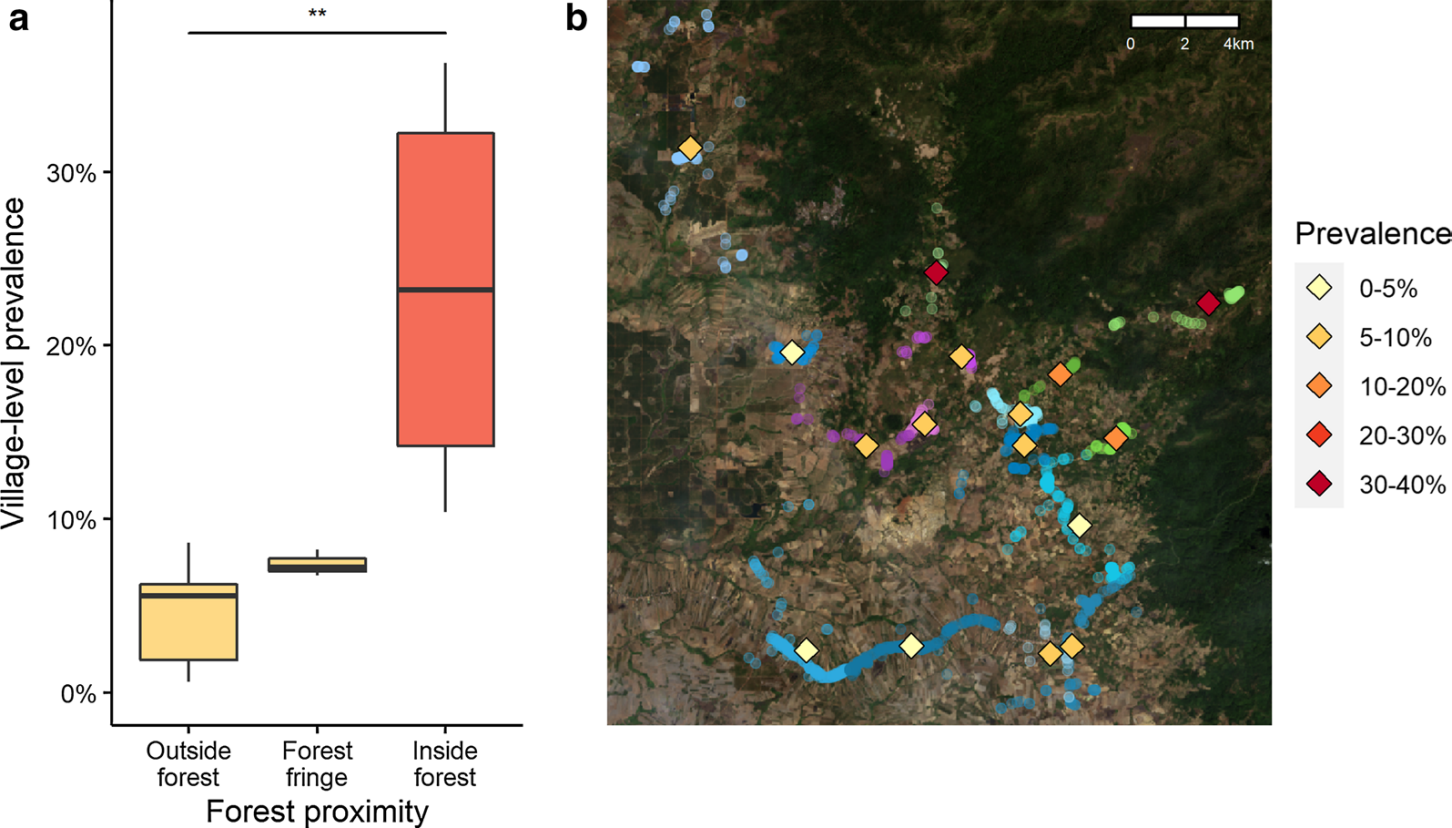
Prevalence of *P. vivax* infection per village (as boxplots by proximity of villages to the forest in **a** and as squares in **b**). Household locations of survey participants in the map background, transparently coloured by village in shades of blue, purple, or green if village is in category “outside forest”, “forest fringe”, or “inside forest”, respectively. Significance asterisk for Kruskal–Wallis test of differences in village-level prevalence

Infection by *Plasmodium* parasite was detected by PCR in 8.3% (349/4200) of participants. The proportion of samples positive for *P. vivax* was 6.4% (268/4200) compared to 3.0% (125/4200) for *P. falciparum* (*p* < 0.001, Table 1). *Plasmodium vivax* was found in 77% (268/349) of all infections compared to 36% (125/349) for *P. falciparum*. Four samples were positive for *P. malariae* mono-infections and none for *P. ovale*. Speciation by PCR was unsuccessful in 4 samples which were analysed as negative. Extrapolation to the entire Kaev Seima population yielded an estimated prevalence of 8.9% for *Plasmodium* infection, 6.8% for *P. vivax*, and 3.3% for *P. falciparum*. Estimated prevalence was highly heterogeneous across the villages, ranging from 0.6% (3/594) to 36.3% (54/152) and from 0% (0/594) to 25.1% (27/106) for *P. vivax* and *P. falciparum*, respectively (both *p* < 0.001, Table 1). Village-level prevalence of *P. vivax* infection was on average higher in villages inside the forest compared with those at the forest fringe and outside the forest (medians 23.2, 7.2, 5.6% respectively, Fig. 2; similarly for *P. falciparum* in Additional file 1: Fig. S2).

**Table 1:** Prevalence of PCR-detected infections

	N	PCR positivity			
		*Plasmodium*	*P. vivax*	*P. falciparum*	Co-infections
Proportion (n) of survey samples	4200	8.3% (349)	6.4% (268)	3.0% (125)	1.1% (48)
Extrapolated population prevalence	10,053	8.9%	6.8%	3.3%	1.3%
Extrapolated prevalence per village					
*Outside forest*					
Trapaingphiae	594	0.6% (3)	0.6% (3)	0% (0)	0% (0)
Chhnaeng	550	2.7% (12)	1.9% (8)	0.9% (4)	0.3% (1)
Oham	333	8.0% (20)	6.2% (16)	2.9% (7)	1.1% (3)
Ohrana	470	5.7% (25)	4.2% (18)	1.8% (8)	0.2% (1)
Sraepreas	228	6.5% (13)	5.6% (11)	1.7% (3)	0.8% (1)
Sraektum	296	13.1% (36)	8.6% (24)	4.8% (13)	0.8% (2)
Lapakhe	71	10.6% (4)	6.7% (3)	7.8% (2)	3.9% (1)
Ohkaunpreas	317	6.9% (21)	5.9% (18)	1.7% (5)	0.7% (2)
Trapaingtouk	201	1.9% (3)	1.2% (2)	0.6% (1)	0% (0)
*Forest fringe*					
Poucha	239	9.7% (24)	6.7% (17)	4.3% (10)	1.4% (3)
Sraeampilkroam	218	11.0% (23)	7.2% (15)	3.8% (8)	0.6% (1)
Sraeampilleu	168	10.6% (17)	8.2% (13)	2.4% (4)	0% (0)
*Inside forest*					
Beng-Gaty	106	37.9% (40)	30.9% (32)	25.1% (27)	18.1% (19)
Ohchra	152	40.4% (60)	36.3% (54)	10.0% (15)	5.9% (9)
Sraelvy	154	18.7% (28)	15.5% (23)	3.9% (6)	1.3% (2)
Ohtrone	103	19.7% (20)	10.4% (11)	12.4% (12)	3.1% (3)

As proportions of positive blood samples or as census population-level estimates by extrapolation via post-sampling weights (raw numbers of positive samples in brackets). 4 positive *P. malariae* samples omitted

### *Plasmodium vivax* infections were least detectable by the health care system

Of all PCR-detected infections, 83.2% (223/268) were sub-microscopic for *P. vivax* compared to 74.4% (93/125) for *P. falciparum* (*p*≈0.056, Table 2). *Plasmodium vivax* infections coincided less often with reported or measured symptoms at the interview than those by *P. falciparum*, with 6.7% (18/268) and 16.8% (21/125), respectively (*p* < 0.01). Only 2.2% (6/268) of all *P. vivax* infections were symptomatic and also positive by RDT or LM (*i.e.* detectable through the Cambodian health care system), less often than for *P. falciparum* (7.2%, 9/125, *p* < 0.05). Asexual parasite stages were detected in all LM^+^ samples for *P. vivax* (37/37) and in most (81.5%, 22/27) for *P. falciparum* (Table 3). In LM^+^ samples, the geometric mean parasite densities were 149.7 parasites/μL and 431.3 parasites/μL (*t*-test: p≈0.07), respectively. While gametocytes were found in only one (2.7%, 1/37) *P. vivax* LM^+^ sample, more than a third (37.0%, 10/27) of LM^+^ samples for *P. falciparum* were gametocyte-positive with a geometric mean of 51.1 gametocytes/μL.

**Table 2:** Detectability of infections through the public health care system

Measure	PCR positivity	
	*P. vivax*	*P. falciparum*
LM^+^	16.8% (45/268)	25.6% (32/125)
Symptoms at interview	6.7% (18/268)	16.8% (21/125)
Symptoms & LM^+^	2.2% (6/268)	4.8% (6/125)
Symptoms & RDT^+^	0.4% (1*/268)	4.0% (5/125)
Symptoms & RDT/LM^+^	2.2% (6/268)	7.2% (9/125)

Proportion (N) of PCR-positive samples that were also detectable by LM, inspection of symptoms, or by testing of symptomatic individuals with LM or RDTs

*Actually RDT^+^ for* P. falciparum* in a co-infection with *P. vivax* as by PCR

**Table 3:** Densities of asexual and sexual stages in LM^+^ samples

	LM positivity	
	*P. vivax*	*P. falciparum*
*Asexual parasitaemia*		
Proportion (N) of LM^+^ samples with detected asexual parasites	100% (37/37)	81.5% (22/27)
Geometric mean [/μl blood]	149.7	431.3
Range [/μl blood]	15.4–22,674.7	14.3–41,724.6
*Gametocytaemia*		
Proportion (N) of LM^+^ samples with gametocytes	2.7% (1/37)	37.0% (10/27)
Geometric mean [/μl blood]	31.9	51.1
Range [/μl blood]	31.9–31.9	15.6–175.0

Geometric mean and range always given from among samples with non-zero densities

### Prevalence was highest in men of working age

**Fig. 3:**
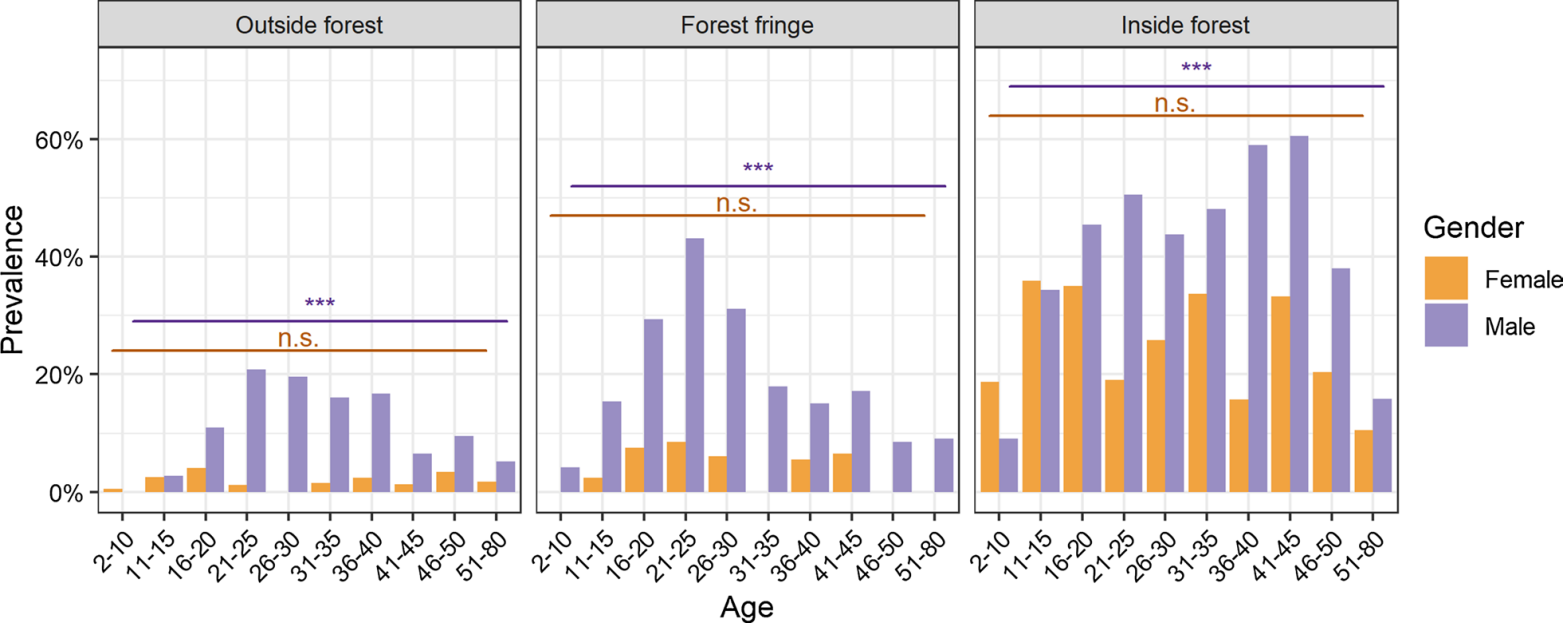
Prevalence of *Plasmodium* infection by age per gender and category of village forest proximity. Significance asterisks for test of differences in prevalence across age groups per gender strata

Estimated prevalence was more than twice as high in men as in women, *i.e.* 10.4% (188/1969) *vs.* 3.6% (80/2231) for *P. vivax* (*p* < 0.001), 4.8% (83/1969) *vs.* 1.9% (42/2231) for *P. falciparum* (*p* < 0.001), and 13.3% (239/1969) *vs.* 5.0% (110/2231) regardless of species (*p* < 0.001). The patterns across age were similarly heterogeneous for both species (Additional file 1: Figure S3). While there was no difference in genus-wide prevalence across age in women, men showed an elevated risk at working age (*p* < 0.001), regardless of the proximity of the village to the forest (Fig. 3). A fitted interaction term of gender and age was significant in the strata of villages outside the forest (*p* < 0.001), but not across those villages at the forest fringe or inside the forest.

### Risk profiles for *P. vivax* and *P. falciparum* infections were similar

Risk of infection was associated with individual covariates for both *P. vivax* and *P. falciparum* in a highly similar fashion (Table 4 for behavioural variables, full list of variables with odds ratios in Additional file 1: Table ST2). For both species, prevalence was associated with work-unrelated overnight travels and highest if those occurred to forest sites (22.4%, 60/268, and 14.9%, 40/268, for *P. vivax* and *P. falciparum*, respectively) and lowest for urban destinations (1.7%, 1/59, and 0%, 0/59, for both *p* < 0.001). Infections of both species were also more prevalent among those who reported work trips to sites in the nearby and deep forest (assessed separately). In particular, a significantly higher prevalence was observed in participants who reported work trips into the deep forest (25.5%, 37/145, and 18.6%, 27/145) compared to those who did not (5.7%, 231/4055, and 2.4%, 98/4055, for *P. vivax* and *P. falciparum*, respectively, *p* < 0.001). Once clustering at household and village-level was taken into account in univariate mixed-effects logistic regression, work in the nearby forest was no longer significantly associated with risk of infection though (Additional file 1: Table ST2). There were fewer infections among those who reported the use of standard protection measures such as sleeping under a bed net (6.1%, 236/3880, and 2.7%, 104/3880, *vs.* 10.0%, 32/320, and 6.6%, 21/320, if no net was used, *p* < 0.01 and *p* < 0.001 for *P. vivax* and *P. falciparum*, respectively). A higher risk in men, at working age, in villages at the forest fringe or inside the forest, and with indicators of lower socio-economic status was also found similarly for both species (Additional file 1: Table ST2).

**Table 4:** Univariate association of infection risk and behavioural covariates

Covariate	N	*P. vivax*		*P. falciparum*	
		Prevalence	*p*-value	Prevalence	*p*-value
*Work-unrelated travels overnight in the last month to…*					
None	3465	4.9% (171)	< 0.001	2.0% (70)	< 0.001
Field sites	90	3.3% (3)		4.4% (4)	
Forest sites	268	22.4% (60)		14.9% (40)	
A village	177	13.6% (24)		5.6% (10)	
A city	59	1.7% (1)		0% (0)	
Unspecified	123	7.3% (9)		0.8% (1)	
*Work in the last two months in…*					
…deep forest					
No	4055	5.7% (231)	< 0.001	2.4% (98)	< 0.001
Yes	145	25.5% (37)		18.6% (27)	
…cassava field					
No	2115	5.6% (118)	< 0.05	2.9% (62)	n.s
Yes	2085	7.2% (150)		3.0% (63)	
No work					
No	3186	7.9% (253)	< 0.001	3.7% (119)	< 0.001
Yes	1014	1.5% (15)		0.6% (6)	
*Slept outdoors last night*					
Indoors	4124	6.2% (256)	< 0.01	2.9% (118)	< 0.01
Outdoors	76	15.8% (12)		9.2% (7)	
*Sprays repellent usually at bedtime*					
No	3668	6.7% (247)	< 0.05	3.2% (116)	< 0.10
Yes	524	4.0% (21)		1.7% (9)	
*Slept under net last night*					
No	320	10.0% (32)	< 0.01	6.6% (21)	< 0.001
Yes	3880	6.1% (236)		2.7% (104)	

Prevalence (n) of PCR-positivity across the strata of those behavioural variables that were statistically significant in univariate logistic regression. “n.s.”: Not significant

### Working in and travelling to the forest were strong risk factors of infection

Behavioural covariates related to activities in the forest were statistically significant in the multivariate model of *Plasmodium* infection (Table 5). Having travelled to forest sites (nearby or deep forest) or having worked in the deep forest independently increased the odds of infection two to three-fold (adjusted odds ratio, aOR, 2.17, *p* < 0.01 and aOR 2.88, *p* < 0.001, respectively). Risk of infection was not significantly attributed to the other behavioural covariates that were included in the model a priori, namely having slept outdoors (aOR 1.99, *p*≈0.08) and under a bed net (aOR 0.99, *p*≈0.96). Risk of infection was increased in males (aOR 3.06, *p* < 0.001) and working age (aOR 7.84 in 21–25 years old compared to children, *p* < 0.001). A fitted interaction term of gender and age improved the model (AIC 1786 *vs.* 1802 without interaction, *p* < 0.001, Additional file 1: Table ST3). Other covariates linked higher socio-economic status with lower odds of infection, such as living in a house with a roof built of relatively high-quality material (aOR 0.50, *p* < 0.01). The covariate on recent information on malaria via TV (aOR 0.37, *p* < 0.01) most likely also acts as a proxy for higher socio-economic status, i.e. being able to afford a TV in the first place.

**Table 5:** Risk factors after multivariate mixed-effects logistic regression for *Plasmodium* infection as detected by PCR

Covariate	N	aOR	95% CI	*p*
Gender				
Female	2231	Reference		< 0.001
Male	1969	3.06	[2.26–4.13]	
Age [years]				
2–10	1054	Reference		< 0.001
11–15	527	4.51	[2.52–8.06]	
16–20	424	7.47	[4.14–13.47]	
21–25	335	7.84	[4.20–14.63]	
26–30	347	8.08	[4.36–14.94]	
31–35	309	7.71	[4.05–14.70]	
36–40	289	7.15	[3.76–13.58]	
41–45	244	5.33	[2.67–10.65]	
46–50	197	4.16	[1.92–8.99]	
51–80	474	2.68	[1.36–5.29]	
Forest proximity of village				
Outside forest	3060	Reference		< 0.001
Forest fringe	625	2.14	[1.02–4.52]	
Inside forest	515	12.47	[6.29–24.71]	
Material of roof				
Grass/leaves	81	0.96	[0.26–3.55]	< 0.01
Tent	79	3.43	[1.36–8.64]	
Corrugated iron	3301	Reference		
Wood planks/cement/tiles	739	0.50	[0.30–0.84]	
Household owns a toilet				
No	2383	Reference		< 0.05
Yes	1816	0.69	[0.48–1.00]	
Household owns buffaloes				
No	3977	Reference		< 0.01
Yes	222	2.11	[1.21–3.69]	
Household head had received information on malaria via TV in the past 3 months				
No	3784	Reference		< 0.01
Yes	415	0.37	[0.16–0.81]	
Insecticides had been sprayed inside the house in the past year				
No	2935	Reference		< 0.01
Yes	1264	0.62	[0.43–0.89]	
Slept outdoors last night				
No	4124	Reference		0.08
Yes	76	1.99	[0.93–4.25]	
Slept under net last night				
No	320	Reference		0.96
Yes	3880	0.99	[0.61–1.59]	
Work-unrelated travels overnight in the last month to…				
None	3465	Reference		< 0.01
Field sites	90	0.63	[0.25–1.59]	
Forest sites	268	2.17	[1.41–3.35]	
A village	177	1.28	[0.75–2.17]	
A city	59	0.27	[0.03–2.13]	
Unspecified	123	1.23	[0.56–2.74]	
Work in the last two months in…				
…deep forest				
No	4055	Reference		< 0.001
Yes	145	2.88	[1.69–4.93]	
…cassava field				
No	2115	Reference		< 0.05
Yes	2085	1.37	[1.01–1.85]	

### Descriptive characterization of the risk factors forest work and forest travels

Work-unrelated overnight travels to forest sites was a predominantly male domain (reported by 10.7%, 211/1969, of the men *vs.* 2.6%, 57/2231, of the women, *p* < 0.001) and particularly frequent in male adolescents (with 24.3%, 70/289, highest in 21–30 years old males, *p* < 0.001). Forest work was independently assessed for sites of the nearby and deep forest. Only for the latter, a statistically significant association with risk was observed. As for travels, they were reported more frequently by men than by women (6.9%, 135/1969, *vs.* 0.4%, 10/2231, *p* < 0.001) and most often by 21–30 years old males (17.6%, 51/289, *p* < 0.001). However, working in the deep forest occurred in both men aged between 8 and 60 years and in 8–43 years old women. Forest travels were reported in 3–62 years old men and 3–63 years old women. Both kind of trips occur usually frequently and of short duration. In 81% (217/268) of the times, the travels to forest sites lasted less than a week. Work trips into the deep forest occurred in 75% (108/145) of the times at least weekly. When going for work several times a month or once a month, the reported duration was also most often less than a week (83%, 15/18, and 53%, 8/15, of the times, respectively). A net was used during overnight work trips into the forest in only 25% (28/113) of the instances. While equally few participants from villages outside the forest, at the forest fringe, and inside the forest reported work in the deep forest (3.4%, 105/3060, 2.9%, 18/625, and 4.3%, 22/515, respectively, *p*≈0.44), travelling to forest sites was reported more often in forest fringe and forest villages (8.6%, 54/625, and 11.5%, 59/515, respectively, compared to 5.1%, 155/3060, outside the forest, *p* < 0.001).

### Residing in a village inside the forest was an independent spatial risk factor

**Fig. 4:**
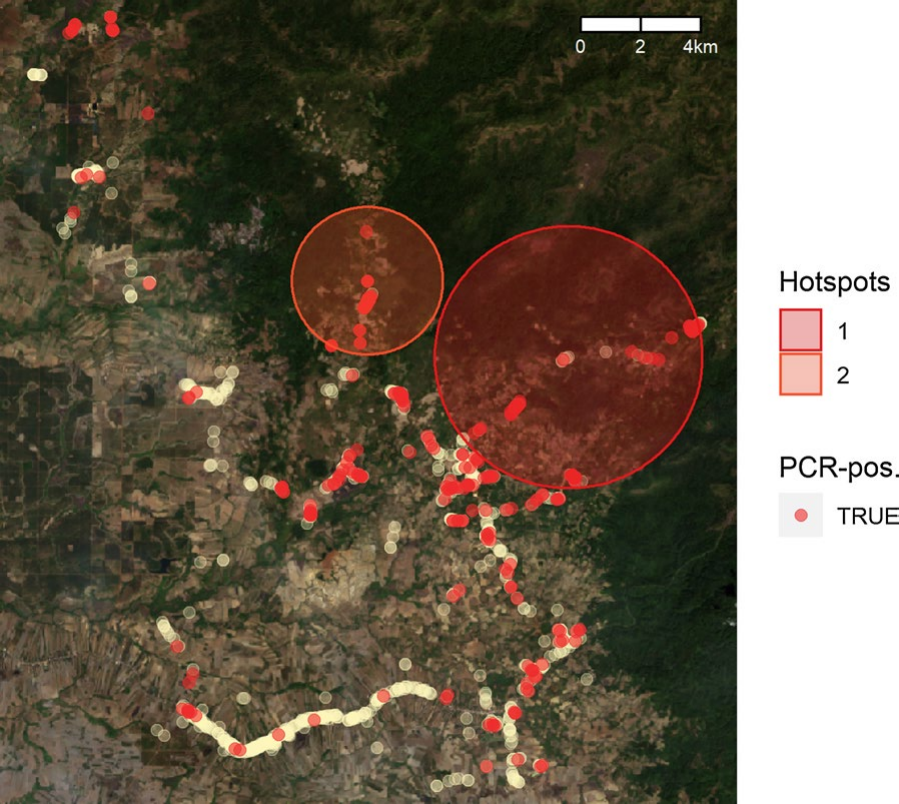
Spatial clusters of *Plasmodium* infection (red-shaded circles) as detected by covariate-adjusted SaTScan analysis. White dots represent household locations in the survey, red dots for PCR-positive participants

Living in a village inside the forest remained associated with risk of infection when adjusting for the significant demographic, socio-economic, and behavioural covariates (aOR 12.47, *p* < 0.001, Table 5). Households from all four villages inside the forest also formed hotspots of *Plasmodium* infection, *i.e.* purely spatial clusters of elevated risk of infection that cannot be explained by the other covariates (Fig. 4, Additional file 1: Tables ST4–ST5).

## Discussion

This is the first detailed study on the prevalence of *Plasmodium* infection and associated risk factors on population-level in Mondulkiri province in Cambodia. The overall prevalence of *Plasmodium* infection is consistent with that observed in the neighbouring province of Ratanakiri [10, 11, 22] or other endemic provinces in the West of the country [16].

*Plasmodium falciparum* has long been the predominant species countrywide [9, 34]. However, following a scale-up in the VMW programmes, a steady decrease in reported *P. falciparum* cases was observed in 2009–2011, while numbers of reported *P. vivax* cases increased [24]. In recent years, cases of both species were reported at an almost equal share [4] and in 2018 *P. vivax* accounted for approximately three quarters of reported cases [2]. That *P. vivax* started to predominate over *P. falciparum* in Cambodia even earlier is suggested by other studies in high-incidence provinces where molecular and serological diagnosis identified a higher prevalence of *P. vivax* than *P. falciparum* infections [11, 12, 15–19], consistent with the presented study in Mondulkiri.

7.2% of the *P. falciparum* infections could be identified by symptoms and a positive result by LM or RDTs, *i.e.* the diagnostics used by the health system, compared to only 2.2% for *P. vivax*. Low sensitivity of RDTs for *P. vivax* is a commonly reported problem [12, 35–38]. In addition, higher proportions of asymptomatic infections for *P. vivax* are regularly observed as the relapses can lead to higher levels of immunity and thus overall lower parasite densities [39, 40]. Data from studies using membrane feeding assays suggest that the densities of asexual stages and gametocytes in the asymptomatic, LM-positive samples in this survey are infectious to mosquitoes [14, 41–45]. It can thus not be ruled out that these subclinical infections contribute to the ongoing transmission in this area.

More asymptomatic infections, lower diagnostic sensitivity for *P. vivax*, high transmissibility, and the lack of radical cure by primaquine all might add up as possible explanations why the control efforts have been less successful against *P. vivax* than against *P. falciparum*. In order to address this high burden of *P. vivax*, Cambodia is now piloting the use of primaquine against *P. vivax* infections in four provinces. While an essential step, the effect of radical cure will be limited without point-of-care diagnostics that are more sensitive for *P. vivax* infections and ideally a test allowing the detection of hypnozoite carriage (irrespective of blood stage parasitaemia). New advances in the use of multiple antigens to detect antibodies as serological markers for recent exposure and thus for potential hypnozoite carriers are promising in this respect [46].

With as many as 40% of the inhabitants infected in the highest-prevalence village in this survey, it may be necessary to complement the current malaria control efforts by active or reactive case detection schemes [47, 48] to reduce the infected (and potentially infectious) reservoir and eventually reach the goal of malaria elimination. Targeting such resource-intensive interventions to high-risk groups will render them significantly more cost-efficient. The similarity of the risk profiles for both *P. falciparum* and *P. vivax* presented here is encouraging as it suggests that such targeted interventions would appropriately address both of the two species.

This study emphasizes the predominant role of forest malaria transmission in explaining elevated risk of infection in remote rural areas of Southeast Asia. Previous cross-sectional surveys have also identified an association of infection risk with either work in the forest [15, 17, 49] or time spent therein [16, 50]. However, this study is unique in retaining them both as statistically significant risk factors in one multivariate model while also adjusting for gender, age, socio-economic proxies, and other behavioural covariates. While work in and travels into the forest are most frequent in adolescent and adult men who are usually considered the forest-related risk group, this behaviour also occurred in women and from children to participants in their sixties and thus actually extends to a much broader range of the population.

On the relatively small spatial scale of 17 villages in an area of 22 × 26 km^2^, the prevalence ranged from less than 1% to above 40% in the dry season. The trend towards higher prevalence in villages at the forest fringe and inside the forest compared to those outside the forest is in line with the widely accepted notion for forest-related mosquito abundance and exposure in Cambodia. Other studies have also identified a higher prevalence in villages at close proximity to the forest [21, 49, 50]. It is apparent that the association of risk with living near or inside the forest was retained in this study even if adjusting for demographic, socio-economic, occupational, and other behavioural differences.

Not more than 60% of infections could be attributed to the strong behavioural, forest-related risk factors. In villages outside the forest or at the forest fringe, most infections indeed occurred in working age men. This predominance of this occupational risk group was much lower in forest fringe villages and entirely lost in villages situated inside the forest. Taking gender and age as proxies for risk-related behaviour and occupational activities, this could indicate that in villages outside the forest, risk can be explained almost completely by such occupational exposure. By contrast, for villages within the forest, the more homogeneous presence of infection across all age groups in both males and females indicates additional exposure inside the village. This is in line with other studies in which household-level risk factors in forested areas were important to explain infection risk [10] or infections clustered in households over 2 years [51] in Ratanakiri, the other Eastern province of Cambodia.

The current WHO report on malaria eradication calls for subnational stratification of intervention programmes [52, 53]. This study suggests that such a stratification of interventions could happen along a gradient of villages inside or outside the forest. Population-oriented interventions such as targeted mass drug administration or test and treat programmes with sensitive molecular or serological diagnostics [46, 54] (together with their costs and risk of overtreatment) could be justified in forest villages. By contrast, approaches targeted at risk groups only may be effective at lower costs for the NMCP and the local population in residential settings outside of the forest.

## Conclusion

Despite a substantial reduction in malaria burden in Cambodia over the last decades, this study demonstrates that pockets of high malaria prevalence persist in the country. Given that the vast majority of infections were asymptomatic, the study strengthens the argument to enhance malaria elimination efforts by measures of (re-)active detection of also asymptomatic infections. *Plasmodium vivax* infections were detected at a higher prevalence than *P. falciparum* infections, more often asymptomatic, and less detectable by RDT and LM. Consequently, novel tools for the identification of hypnozoite carriers could play a key role in the presented setting as well as the roll-out of routine radical cure treatment. The study corroborates the notion of forest malaria in the Greater Mekong Subregion by demonstrating the independent association of travels to the forest, of forest work, and of living in close proximity to forest with malaria infection risk. However, the study also demonstrates that infection risk is less confined to forest-goers in sub-populations that already live in forested areas and suggests within-village transmission therein. A focus of interventions solely on forest-goers could thus be insufficient to reach the goal of nation-wide malaria elimination in due time. In the light of the presented results, interventions could be targeted at whole villages inside the forest, and targeted at stratified risk groups for villages outside the forest.

## Supplementary information

**Additional file 1.** Additional tables ST1–ST5 and additional figures S1–S3.

## Data Availability

The de-identified dataset analysed for this study is being made publicly available in ClinEpiDB repository, https://clinepidb.org/ce/app/. In the meantime, it is available from the corresponding author on reasonable request.

## Abbreviations

ACT: Artemisinin-based combination therapy
AIC: Akaike Information Criterion
aOR: Adjusted odds ratio
G6PD: Glucose-6-phosphate dehydrogenase
GPS: Global positioning system
K^+^EDTA: Potassium ethylenediaminetetraacetic acid
LLIN: Long-lasting insecticidal net
LM: Light microscopy
MMW: Mobile malaria worker
N: Sample size
NMCP: National malaria control programme
PCR: Polymerase chain reaction
RDT: Rapid diagnostic test
VMW: Village malaria worker
WHO: World Health Organization

## References

[1] National Center for Parasitology, Entomology and Malaria Control (CNM). Annual Report 2018. Phnom Penh; 2019.

[2] WHO (2019). World malaria report 2019.

[3] WHO (2019). Countries of the Greater Mekong zero in on falciparum malaria.

[4] National Center for Parasitology, Entomology and Malaria Control (CNM). Annual Report 2017. Phnom Penh; 2018.

[5] Ministry of Health, Kingdom of Cambodia. Cambodia Malaria Elimination Action Framework 2016–2020. Phnom Penh; 2016.

[6] Duru V, Witkowski B, Ménard D (2016). *Plasmodium falciparum* resistance to artemisinin derivatives and piperaquine: a major challenge for malaria elimination in Cambodia. Am J Trop Med Hyg.

[7] President’s malaria initiative. Malaria Operational Plan FY 2018, Cambodia. Phnom Penh; 2018

[8] National Center for Parasitology, Entomology and Malaria Control (CNM). National Strategic Plan for Elimination of Malaria in Cambodia (2011–2025). Phnom Penh; 2011.

[9] Lek D, Popovici J, Ariey F, Vinjamuri SB, Meek S, Bruce J (2016). National malaria prevalence in Cambodia: microscopy versus polymerase chain reaction estimates. Am J Trop Med Hyg.

[10] Bannister-Tyrrell M, Srun S, Sluydts V, Gryseels C, Mean V, Kim S (2018). Importance of household-level risk factors in explaining micro-epidemiology of asymptomatic malaria infections in Ratanakiri Province, Cambodia. Sci Rep.

[11] Durnez L, Pareyn M, Mean V, Kim S, Khim N, Menard D (2018). Identification and characterization of areas of high and low risk for asymptomatic malaria infections at sub-village level in Ratanakiri, Cambodia. Malar J.

[12] Imwong M, Nguyen TN, Tripura R, Peto TJ, Lee SJ, Lwin KM (2015). The epidemiology of subclinical malaria infections in South-East Asia: findings from cross-sectional surveys in Thailand-Myanmar border areas, Cambodia, and Vietnam. Malar J.

[13] Vantaux A, Samreth R, Piv E, Khim N, Kim S, Berne L (2018). Contribution to malaria transmission of symptomatic and asymptomatic parasite carriers in Cambodia. J Infect Dis.

[14] Kiattibutr K, Roobsoong W, Sriwichai P, Saeseu T, Rachaphaew N, Suansomjit C (2017). Infectivity of symptomatic and asymptomatic *Plasmodium vivax* infections to a Southeast Asian vector, *Anopheles dirus*. Int J Parasitol.

[15] Kerkhof K, Sluydts V, Heng S, Kim S, Pareyn M, Willen L (2016). Geographical patterns of malaria transmission based on serological markers for falciparum and vivax malaria in Ratanakiri, Cambodia. Malar J.

[16] Tripura R, Peto TJ, Veugen CC, Nguon C, Davoeung C, James N (2017). Submicroscopic *Plasmodium* prevalence in relation to malaria incidence in 20 villages in western Cambodia. Malar J.

[17] Parker DM, Tripura R, Peto TJ, Maude RJ, Nguon C, Chalk J (2017). A multi-level spatial analysis of clinical malaria and subclinical *Plasmodium* infections in Pailin Province, Cambodia. Heliyon.

[18] Tripura R, Peto TJ, Chalk J, Lee SJ, Sirithiranont P, Nguon C (2016). Persistent *Plasmodium falciparum* and *Plasmodium vivax* infections in a western Cambodian population: implications for prevention, treatment and elimination strategies. Malar J.

[19] Bosman P, Stassijns J, Nackers F, Canier L, Kim N, Khim S (2014). *Plasmodium* prevalence and artemisinin-resistant falciparum malaria in Preah Vihear Province, Cambodia: a cross-sectional population-based study. Malar J.

[20] Cook J, Speybroeck N, Sochanta T, Somony H, Sokny M, Claes F (2012). Sero-epidemiological evaluation of changes in *Plasmodium falciparum* and *Plasmodium vivax* transmission patterns over the rainy season in Cambodia. Malar J.

[21] Incardona S, Vong S, Chiv L, Lim P, Nhem S, Sem R (2007). Large-scale malaria survey in Cambodia: novel insights on species distribution and risk factors. Malar J.

[22] Sluydts V, Heng S, Coosemans M, Van Roey K, Gryseels C, Canier L (2014). Spatial clustering and risk factors of malaria infections in Ratanakiri Province, Cambodia. Malar J.

[23] Steenkeste N, Rogers WO, Okell L, Jeanne I, Incardona S, Duval L (2010). Sub-microscopic malaria cases and mixed malaria infection in a remote area of high malaria endemicity in Rattanakiri province, Cambodia: implication for malaria elimination. Malar J.

[24] Maude RJ, Nguon C, Ly P, Bunkea T, Ngor P, Canavati de la Torre SE (2014). Spatial and temporal epidemiology of clinical malaria in Cambodia 2004–2013. Malar J..

[25] Asian Development Bank. Indigenous peoples/ethnic minorities and poverty reduction: Cambodia. Phnom Penh; 2002.

[26] Moul P, Seng S (2012). Country Technical Note on Indigenous Peoples’ Issues: Kingdom of Cambodia.

[27] Canier L, Khim N, Kim S, Sluydts V, Heng S, Dourng D (2013). An innovative tool for moving malaria PCR detection of parasite reservoir into the field. Malar J.

[28] Pepey A, Souris M, Vantaux A, Morand S, Lek D, Mueller I (2020). Studying land cover changes in a malaria-endemic Cambodian district: considerations and constraints. Remote Sens.

[29] Kulldorff M (1997). A spatial scan statistic. Commun Stat Theory Methods.

[30] Harris PA, Taylor R, Thielke R, Payne J, Gonzalez N, Conde JG (2009). Research electronic data capture (REDCap)–a metadata-driven methodology and workflow process for providing translational research informatics support. J Biomed Inform.

[31] Harris PA, Taylor R, Minor BL, Elliott V, Fernandez M, O’Neal L (2019). The REDCap consortium: Building an international community of software platform partners. J Biomed Inform.

[32] R Core Team. R: A Language and Environment for Statistical Computing. Vienna, Austria: R Foundation for Statistical Computing; 2019. https://www.R-project.org/

[33] Kulldorff M, Information Management Services, Inc. SaTScan^TM^ v9.6: Software for the spatial and space-time scan statistics. 2018. www.satscan.org

[34] World Health Organization (2018). World malaria report 2018.

[35] Cho SJ, Lee J, Lee HJ, Jo H-Y, Sinniah M, Kim H-Y (2016). A novel malaria Pf/Pv Ab rapid diagnostic test using a differential diagnostic marker identified by network biology. Int J Biol Sci.

[36] Landier J, Parker DM, Thu AM, Lwin KM, Delmas G, Nosten FH (2018). Effect of generalised access to early diagnosis and treatment and targeted mass drug administration on *Plasmodium falciparum* malaria in Eastern Myanmar: an observational study of a regional elimination programme. Lancet.

[37] Tadesse E, Workalemahu B, Shimelis T (2016). Diagnostic performance evaluation of the SD Bioline Malaria Antigen AG Pf/Pan test (05FK60) in a malaria endemic area of southern Ethiopia. Rev Inst Med Trop São Paulo.

[38] Kim SH, Nam M-H, Roh KH, Park HC, Nam DH, Park GH (2008). Evaluation of a rapid diagnostic test specific for *Plasmodium vivax*. Trop Med Int Health.

[39] White NJ (2011). Determinants of relapse periodicity in *Plasmodium vivax* malaria. Malar J.

[40] Olliaro PL, Barnwell JW, Barry A, Mendis K, Mueller I, Reeder JC (2016). Implications of *Plasmodium vivax* biology for control, elimination, and research. Am J Trop Med Hyg.

[41] Tadesse FG, Slater HC, Chali W, Teelen K, Lanke K, Belachew M (2018). The relative contribution of symptomatic and asymptomatic *Plasmodium vivax* and *Plasmodium falciparum* infections to the infectious reservoir in a low-endemic setting in Ethiopia. Clin Infect Dis.

[42] Churcher TS, Bousema T, Walker M, Drakeley C, Schneider P, Ouédraogo AL (2013). Predicting mosquito infection from *Plasmodium falciparum* gametocyte density and estimating the reservoir of infection. eLife.

[43] Ouédraogo AL, Bousema T, Schneider P, de Vlas SJ, Ilboudo-Sanogo E, Cuzin-Ouattara N (2009). Substantial contribution of submicroscopical *Plasmodium falciparum* gametocyte carriage to the infectious reservoir in an area of seasonal transmission. PLoS ONE.

[44] Gonçalves BP, Kapulu MC, Sawa P, Guelbéogo WM, Tiono AB, Grignard L (2017). Examining the human infectious reservoir for *Plasmodium falciparum* malaria in areas of differing transmission intensity. Nat Commun.

[45] Da DF, Churcher TS, Yerbanga RS, Yaméogo B, Sangaré I, Ouedraogo JB (2015). Experimental study of the relationship between *Plasmodium* gametocyte density and infection success in mosquitoes; implications for the evaluation of malaria transmission-reducing interventions. Exp Parasitol.

[46] Longley RJ, White MT, Takashima E, Brewster J, Morita M, Harbers M (2020). Development and validation of serological markers for detecting recent *Plasmodium vivax* infection. Nat Med.

[47] Rossi G, Vernaeve L, Van den Bergh R, Nguon C, Debackere M, Abello Peiri C (2018). Closing in on the reservoir: proactive case detection in high-risk groups as a strategy to detect *Plasmodium falciparum* asymptomatic carriers in Cambodia. Clin Infect Dis.

[48] Lover AA, Dantzer E, Hongvanthong B, Chindavongsa K, Welty S, Reza T (2018). Prevalence and risk factors for asymptomatic malaria and genotyping of glucose 6-phosphate (G6PD) deficiencies in a vivax-predominant setting, Lao PDR: implications for sub-national elimination goals. Malar J.

[49] Dysoley L, Kaneko A, Eto H, Mita T, Socheat D, Börkman A (2008). Changing patterns of forest malaria among the mobile adult male population in Chumkiri District, Cambodia. Acta Trop.

[50] National Center for Parasitology, Entomology and Malaria Control (CNM). Cambodia malaria survey 2013. Phnom Penh; 2013.

[51] Bannister-Tyrrell M, Krit M, Sluydts V, Tho S, Sokny M, Mean V (2019). Households or hotspots? Defining intervention targets for malaria elimination in Ratanakiri province, Eastern Cambodia. J Infect Dis.

[52] Strategic Advisory Group on Malaria Eradication (2020). Malaria eradication: benefits, future scenarios & feasibility.

[53] Stresman G, Bousema T, Cook J (2019). Malaria hotspots: is there epidemiological evidence for fine-scale spatial targeting of interventions?. Trends Parasitol.

[54] Lek D, Callery JJ, Nguon C, Debackere M, Sovannaroth S, Tripura R (2020). Tools to accelerate falciparum malaria elimination in Cambodia: a meeting report. Malar J.

