# Supplementary material for: Forest malaria in Cambodia: the occupational and spatial clustering of Plasmodium vivax and Plasmodium falciparum infection risk in a cross-sectional survey in Mondulkiri province, Cambodia
Source: Malar J. 2020 Nov 19;19:413. doi: 10.1186/s12936-020-03482-4 (PMC7678315; doi:10.1186/s12936-020-03482-4)
Supplement: Supplementary file 1 — Additional file 1. Additional tables ST1–ST5 and additional figures S1–S3. [file 12936_2020_3482_MOESM1_ESM.docx]

**Supplementary Material**

**Table ST1** Sampling scheme across villages via linearly decreasing sampling weights with increasing village size in order to oversample smaller villages. 250 as minimal sample size if village was not smaller already.

| **Village** | **Size** | **Status for random selection** | **Proportion selected for random sampling** | **N finally enrolled** | **Proportion of village size enrolled** |
| --- | --- | --- | --- | --- | --- |
| Trapaingphiae | 1873 | Weights linearly decreasing with larger village size | 49.8% | 594 | 31.7% |
| Chhnaeng | 1696 |  | 50.9% | 550 | 32.4% |
| Oham | 1364 |  | 52.9% | 333 | 24.4% |
| Ohrana | 1217 |  | 53.7% | 470 | 38.6% |
| Sraepreas | 711 |  | 56.7% | 228 | 32.1% |
| Sraektum | 571 |  | 57.6% | 296 | 51.8% |
| Lapakhe | 416 | 250 is more than linearly decreasing weights would suggest, thus oversampled | 60.1% | 71 | 17.1% |
| Ohkaunpreas | 383 |  | 46.0%* | 317 | 82.8% |
| Poucha | 367 |  | 68.1% | 239 | 65.1% |
| Trapaingtouk | 268 |  | 93.3% | 201 | 75.0% |
| Sraeampilkroam | 258 |  | 96.9% | 218 | 84.4% |
| Gaty | 258 |  | 96.9% | 95 | 36.8% |
| Sraeampilleu | 177 | <250, thus fully sampled | 100% | 168 | 94.9% |
| Sraelvy | 159 |  | 100% | 154 | 96.9% |
| Ohchra | 157 |  | 100% | 152 | 96.8% |
| Ohtrone | 124 |  | 100% | 103 | 83.1% |
| Beng | 54 |  | 100% | 11 | 20.4% |

* Weights and random selection were calculated based on village size of 176 identified initially during the census. Later, the field team realized to have missed a part of the village and thus appended the census.

**Table ST2** Full list of covariates and their association with prevalence (“pos.”) and risk of infection (as detected by PCR) for P. vivax and P. falciparum. Statistical significance of the covariate based on prevalence by χ^2^ test (or Fisher’s exact test if required) and based on univariate logistic regression with random intercepts for households and villages by likelihood ratio test (LRT). “n.s.”: Not significant (p≥0.05).

| **Covariate** | | **N** | ***P. vivax*** | | | | |  | ***P. falciparum*** | | | | |
| --- | --- | --- | --- | --- | --- | --- | --- | --- | --- | --- | --- | --- | --- |
|  |  |  | **pos. (n)** | ***p (χ^2^)*** | **OR** | **95% CI** | ***p (LRT)*** |  | **pos. (n)** | ***p (χ^2^)*** | **OR** | **95% CI** | ***p (LRT)*** |
| Gender | Female | 2231 | 3.6% (80) | <0.001 | Reference | | |  | 1.9% (42) | <0.001 | Reference | | |
|  | Male | 1969 | 9.5% (188) |  | 3.45 | [2.60-4.57] | <0.001 |  | 4.2% (83) |  | 2.69 | [1.78-4.07] | <0.001 |
| Age [years] | 2-10 | 1054 | 1.5% (16) | <0.001 | Reference | | |  | 0.7% (7) | <0.001 | Reference | | |
|  | 11-15 | 527 | 7.0% (37) |  | 4.41 | [2.43-8.01] | <0.001 |  | 3.0% (16) |  | 4.44 | [1.74-11.34] | <0.001 |
|  | 16-20 | 424 | 11.6% (49) |  | 8.16 | [4.58-14.56] |  |  | 3.8% (16) |  | 5.25 | [2.04-13.48] |  |
|  | 21-25 | 335 | 10.1% (34) |  | 8.73 | [4.74-16.06] |  |  | 5.4% (18) |  | 10.18 | [4.00-25.88] |  |
|  | 26-30 | 347 | 10.1% (35) |  | 9.11 | [4.96-16.74] |  |  | 4.9% (17) |  | 8.49 | [3.30-21.81] |  |
|  | 31-35 | 309 | 8.4% (26) |  | 6.73 | [3.54-12.77] |  |  | 4.5% (14) |  | 9.21 | [3.48-24.33] |  |
|  | 36-40 | 289 | 9.0% (26) |  | 6.51 | [3.40-12.43] |  |  | 4.2% (12) |  | 7.48 | [2.76-20.26] |  |
|  | 41-45 | 244 | 6.6% (16) |  | 4.61 | [2.25-9.41] |  |  | 5.3% (13) |  | 8.66 | [3.20-23.45] |  |
|  | 46-50 | 197 | 6.1% (12) |  | 4.73 | [2.20-10.18] |  |  | 3.6% (7) |  | 6.40 | [2.07-19.80] |  |
|  | 51-80 | 474 | 3.6% (17) |  | 2.88 | [1.46-5.70] |  |  | 1.1% (5) |  | 1.76 | [0.52-5.93] |  |
| Forest proximity of village | Outside forest | 3060 | 3.4% (103) | <0.001 | Reference | | |  | 1.4% (43) | <0.001 | Reference | | |
|  | Forest fringe | 625 | 7.2% (45) |  | 2.43 | [1.03-5.73] | <0.001 |  | 3.5% (22) |  | 2.60 | [1.00-6.75] | <0.001 |
|  | Inside forest | 515 | 23.3% (120) |  | 9.23 | [4.28-19.91] |  |  | 11.7% (60) |  | 16.49 | [6.96-39.05] |  |
| Material of floor | Else | 3578 | 7.0% (249) | <0.001 | Reference | | |  | 3.2% (116) | <0.05 | Reference | | |
|  | Cement/tiles | 622 | 3.1% (19) |  | 0.59 | [0.35-0.97] | <0.05 |  | 1.4% (9) |  | 0.64 | [0.30-1.38] | n.s. |
| Material of walls | No wall/tent | 65 | 13.8% (9) | <0.01 | 2.95 | [1.29-6.76] |  |  | 9.2% (6) | <0.01 | 5.45 | [1.79-16.61] |  |
|  | Wooden | 3802 | 6.5% (248) |  | Reference | | |  | 3.1% (116) |  | Reference | | |
|  | Iron sheets | 217 | 4.6% (10) |  | 1.04 | [0.53-2.03] | n.s. |  | 1.4% (3) |  | 0.66 | [0.19-2.26] | <0.01 |
|  | Cement/bricks | 116 | 0.9% (1) |  | 0.27 | [0.04-1.72] |  |  | 0% (0) |  | No cases | | |
| Material of roof | Grass/leaves | 81 | 3.7% (3) | <0.001 | 1.54 | [0.47-5.08] | <0.01 |  | 1.2% (1) | <0.01 | 1.00 | [0.12-8.30] | n.s. |
|  | Tent | 79 | 12.7% (10) |  | 3.93 | [1.60-9.69] |  |  | 2.5% (2) |  | 1.62 | [0.29-8.87] |  |
|  | Corrugated iron | 3301 | 7.0% (232) |  | Reference | | |  | 3.4% (113) |  | Reference | | |
|  | Wood/cement/tiles | 739 | 3.1% (23) |  | 0.60 | [0.38-0.96] |  |  | 1.2% (9) |  | 0.46 | [0.22-1.00] |  |
| Water source | Surface/rain water | 303 | 9.9% (30) | <0.001 | 1.06 | [0.65-1.74] |  |  | 3.6% (11) | <0.001 | 0.85 | [0.39-1.84] |  |
|  | Well | 2011 | 7.6% (152) |  | Reference | | |  | 3.7% (74) |  | Reference | | |
|  | Tap | 57 | 8.8% (5) |  | 1.46 | [0.49-4.33] | n.s. |  | 8.8% (5) |  | 4.11 | [1.09-15.48] | n.s. |
|  | Bottled water | 1828 | 4.4% (81) |  | 0.79 | [0.57-1.10] |  |  | 1.9% (35) |  | 0.80 | [0.48-1.33] |  |
| Toilet in house | No toilet | 2383 | 8.2% (195) | <0.001 | Reference | | |  | 4.1% (97) | <0.001 | Reference | | |
|  | Any type | 1816 | 4.0% (73) |  | 0.67 | [0.47-0.94] | <0.05 |  | 1.5% (28) |  | 0.56 | [0.33-0.96] | <0.05 |
| Household head attended school | No | 1264 | 8.5% (107) | <0.001 | Reference | | |  | 4.2% (53) | <0.01 | Reference | | |
|  | Yes | 2935 | 5.5% (161) |  | 0.83 | [0.62-1.10] | n.s. |  | 2.5% (72) |  | 0.70 | [0.45-1.07] | n.s. |
| Main income for households | Rubber plantation | 95 | 1.1% (1) | <0.05 | 0.31 | [0.05-2.05] |  |  | 1.1% (1) | n.s. | 0.78 | [0.09-6.52] |  |
|  | Farming | 3829 | 6.7% (256) |  | Reference | | |  | 3.1% (120) |  | Reference | | |
|  | Wood logging | 73 | 9.6% (7) |  | 1.85 | [0.79-4.34] |  |  | 4.1% (3) |  | 1.56 | [0.43-5.72] |  |
|  | Driving | 22 | 9.1% (2) |  | 2.23 | [0.45-11.08] | <0.05 |  | 4.5% (1) |  | 1.96 | [0.18-20.97] | n.s. |
|  | Workmanship | 50 | 2.0% (1) |  | 0.56 | [0.08-3.70] |  |  | 0% (0) |  | No cases | | |
|  | Selling/service | 130 | 0.8% (1) |  | 0.15 | [0.02-0.94] |  |  | 0% (0) |  | No cases | | |
| Household owns |  |  |  |  |  |  |  |  |  |  |  |  |  |
| …chicken | No | 1088 | 5.0% (54) | <0.05 | Reference | | |  | 2.5% (27) | n.s. | Reference | | |
|  | Yes | 3111 | 6.9% (214) |  | 1.12 | [0.80-1.57] | n.s. |  | 3.2% (98) |  | 1.04 | [0.63-1.73] | n.s. |
| …dogs | No | 1585 | 4.9% (77) | <0.01 | Reference | | |  | 2.5% (39) | n.s. | Reference | | |
|  | Yes | 2614 | 7.3% (191) |  | 1.25 | [0.92-1.70] | n.s. |  | 3.3% (86) |  | 1.02 | [0.64-1.61] | n.s. |
| …pigs | No | 3107 | 4.0% (123) | <0.001 | Reference | | |  | 1.7% (54) | <0.001 | Reference | | |
|  | Yes | 1092 | 13.3% (145) |  | 1.40 | [0.98-1.99] | n.s. |  | 6.5% (71) |  | 1.53 | [0.89-2.61] | n.s. |
| …cows | No | 3451 | 5.0% (172) | <0.001 | Reference | | |  | 2.2% (77) | <0.001 | Reference | | |
|  | Yes | 748 | 12.8% (96) |  | 1.45 | [1.02-2.06] | <0.05 |  | 6.4% (48) |  | 1.61 | [0.97-2.68] | n.s. |
| …buffaloes | No | 3977 | 5.9% (234) | <0.001 | Reference | | |  | 2.7% (109) | <0.001 | Reference | | |
|  | Yes | 222 | 15.3% (34) |  | 1.90 | [1.12-3.20] | <0.05 |  | 7.2% (16) |  | 1.98 | [0.94-4.16] | n.s. |
| …ducks | No | 3938 | 6.6% (259) | <0.10 | Reference | | |  | 2.9% (115) | n.s. | Reference | | |
|  | Yes | 261 | 3.4% (9) |  | 0.80 | [0.40-1.61] | n.s. |  | 3.8% (10) |  | 1.98 | [0.90-4.37] | n.s. |
| …cats | No | 3687 | 6.6% (244) | n.s. | Reference | | |  | 3.2% (118) | <0.05 | Reference | | |
|  | Yes | 512 | 4.7% (24) |  | 0.83 | [0.49-1.39] | n.s. |  | 1.4% (7) |  | 0.58 | [0.24-1.40] | n.s. |
| Household head had received information on malaria in the past 3 months via |  |  |  |  |  |  |  |  |  |  |  |  |  |
| …TV | No | 3784 | 6.9% (262) | <0.001 | Reference | | |  | 3.2% (122) | <0.01 | Reference | | |
|  | Yes | 415 | 1.4% (6) |  | 0.31 | [0.14-0.70] | <0.01 |  | 0.7% (3) |  | 0.39 | [0.11-1.32] | n.s. |
| …household visit | No | 3983 | 6.2% (248) | n.s. | Reference | | |  | 2.9% (114) | <0.10 | Reference | | |
|  | Yes | 216 | 9.3% (20) |  | 0.76 | [0.42-1.38] | n.s. |  | 5.1% (11) |  | 1.20 | [0.54-2.64] | n.s. |
| Insecticides had been sprayed inside the house in the past year | No | 2935 | 7.3% (214) | <0.001 | Reference | | |  | 3.6% (106) | <0.001 | Reference | | |
|  | Yes | 1264 | 4.3% (54) |  | 0.60 | [0.43-0.84] | <0.01 |  | 1.5% (19) |  | 0.40 | [0.23-0.71] | <0.001 |
| Work-unrelated travels overnight in the last month to… | None | 3465 | 4.9% (171) | <0.001 | Reference | | |  | 2.0% (70) | <0.001 | Reference | | |
|  | Field sites | 90 | 3.3% (3) |  | 0.81 | [0.26-2.51] |  |  | 4.4% (4) |  | 2.61 | [0.82-8.36] |  |
|  | Forest sites | 268 | 22.4% (60) |  | 5.47 | [3.73-8.01] | <0.001 |  | 14.9% (40) |  | 8.38 | [4.96-14.18] | <0.001 |
|  | A village | 177 | 13.6% (24) |  | 2.79 | [1.68-4.61] |  |  | 5.6% (10) |  | 2.37 | [1.08-5.17] |  |
|  | A city | 59 | 1.7% (1) |  | 0.44 | [0.07-2.85] |  |  | 0% (0) |  | No cases | | |
|  | Unspecified | 123 | 7.3% (9) |  | 2.14 | [1.03-4.42] |  |  | 0.8% (1) |  | 0.50 | [0.06-3.94] |  |
| Work in the last two months in… |  |  |  |  |  |  |  |  |  |  |  |  |  |
| …nearby forest | No | 4053 | 6.2% (252) | <0.05 | Reference | | |  | 2.9% (116) | <0.05 | Reference | | |
|  | Yes | 147 | 10.9% (16) |  | 1.37 | [0.77-2.44] | n.s. |  | 6.1% (9) |  | 1.42 | [0.64-3.15] | n.s. |
| …deep forest | No | 4055 | 5.7% (231) | <0.001 | Reference | | |  | 2.4% (98) | <0.001 | Reference | | |
|  | Yes | 145 | 25.5% (37) |  | 8.03 | [4.83-13.35] | <0.001 |  | 18.6% (27) |  | 10.75 | [5.81-19.89] | <0.001 |
| …rubber plantations | No | 3904 | 6.8% (264) | <0.001 | Reference | | |  | 3.2% (124) | <0.01 | Reference | | |
|  | Yes | 296 | 1.4% (4) |  | 0.60 | [0.22-1.61] | n.s. |  | 0.3% (1) |  | 0.26 | [0.03-2.14] | n.s. |
| …cashew nut plantations | No | 3053 | 5.2% (158) | <0.001 | Reference | | |  | 2.5% (77) | <0.01 | Reference | | |
|  | Yes | 1147 | 9.6% (110) |  | 1.10 | [0.82-1.48] | n.s. |  | 4.2% (48) |  | 0.90 | [0.58-1.40] | n.s. |
| …cassava field | No | 2115 | 5.6% (118) | <0.05 | Reference | | |  | 2.9% (62) | n.s. | Reference | | |
|  | Yes | 2085 | 7.2% (150) |  | 1.71 | [1.28-2.28] | <0.001 |  | 3.0% (63) |  | 1.61 | [1.03-2.50] | <0.05 |
| …rice field | No | 3872 | 6.1% (238) | <0.05 | Reference | | |  | 2.7% (106) | <0.01 | Reference | | |
|  | Yes | 328 | 9.1% (30) |  | 0.75 | [0.48-1.19] | n.s. |  | 5.8% (19) |  | 1.19 | [0.65-2.19] | n.s. |
| No work | No | 3186 | 7.9% (253) | <0.001 | Reference | | |  | 3.7% (119) | <0.001 | Reference | | |
|  | Yes | 1014 | 1.5% (15) |  | 0.24 | [0.14-0.40] | <0.001 |  | 0.6% (6) |  | 0.23 | [0.10-0.53] | <0.001 |
| Having slept outside last night | Indoors | 4124 | 6.2% (256) | <0.01 | Reference | | |  | 2.9% (118) | <0.01 | Reference | | |
|  | Outdoors | 76 | 15.8% (12) |  | 3.11 | [1.54-6.30] | <0.01 |  | 9.2% (7) |  | 2.97 | [1.14-7.74] | <0.05 |
| Sprays repellent usually at bedtime | No | 3668 | 6.7% (247) | <0.05 | Reference | | |  | 3.2% (116) | <0.10 | Reference | | |
|  | Yes | 524 | 4.0% (21) |  | 0.62 | [0.38-1.01] | n.s. |  | 1.7% (9) |  | 0.60 | [0.28-1.27] | n.s. |
| Slept under net last night | No | 320 | 10.0% (32) | <0.01 | Reference | | |  | 6.6% (21) | <0.001 | Reference | | |
|  | Yes | 3880 | 6.1% (236) |  | 0.46 | [0.30-0.71] | <0.01 |  | 2.7% (104) |  | 0.32 | [0.18-0.56] | <0.001 |
| Antimalarials in the last 2 months | No | 3978 | 6.0% (238) | <0.001 | Reference | | |  | 2.3% (91) | <0.001 | Reference | | |
|  | Yes | 211 | 14.2% (30) |  | 1.73 | [1.10-2.73] | <0.05 |  | 16.1% (34) |  | 5.98 | [3.52-10.15] | <0.001 |
| Felt sick at interview | No | 4017 | 6.4% (258) | n.s. | Reference | | |  | 2.8% (114) | <0.05 | Reference | | |
|  | Yes | 170 | 5.9% (10) |  | 0.98 | [0.51-1.91] | n.s. |  | 6.5% (11) |  | 2.47 | [1.19-5.14] | <0.05 |
| Felt feverish at interview | No | 3930 | 6.4% (252) | n.s. | Reference | | |  | 2.7% (108) | <0.001 | Reference | | |
|  | Yes | 249 | 6.4% (16) |  | 0.88 | [0.51-1.51] | n.s. |  | 6.8% (17) |  | 2.39 | [1.31-4.38] | <0.01 |
| Axillary temperature | <37.5°C | 3917 | 6.4% (250) | n.s. | Reference | | |  | 2.7% (106) | <0.001 | Reference | | |
|  | ≥37.5°C | 273 | 5.5% (15) |  | 0.72 | [0.41-1.24] | n.s. |  | 7.0% (19) |  | 2.40 | [1.34-4.28] | <0.01 |

**Table ST3** Fixed effects parameters of multivariate logistic regression model for Plasmodium spp. infection as detected by PCR, including a gender-age interaction term.

| **Covariate** |  | **β** | **Std. error** | ***p*** |
| --- | --- | --- | --- | --- |
| (Intercept) |  | -4.67888 | 0.46687 |  |
| Gender | Female | Reference | | <0.001 |
|  | Male | -0.47247 | 0.46214 |  |
| Age [years] alone | 2-10 | Reference | |  |
|  | 11-15 | 1.06923 | 0.40483 |  |
|  | 16-20 | 1.23856 | 0.40158 |  |
|  | 21-25 | 0.60961 | 0.49079 |  |
|  | 26-30 | 0.46972 | 0.50472 |  |
|  | 31-35 | 0.91935 | 0.49992 |  |
|  | 36-40 | 0.32925 | 0.51226 |  |
|  | 41-45 | 0.81629 | 0.52053 |  |
|  | 46-50 | 0.57800 | 0.63309 |  |
|  | 51-80 | -0.19417 | 0.57775 |  |
| Age [years] in men (gender-age interaction) | 2-10 | Reference | |  |
|  | 11-15 | 0.68542 | 0.58371 |  |
|  | 16-20 | 1.31719 | 0.58402 |  |
|  | 21-25 | 2.43349 | 0.65292 |  |
|  | 26-30 | 2.63857 | 0.66262 |  |
|  | 31-35 | 1.92763 | 0.66384 |  |
|  | 36-40 | 2.74284 | 0.67905 |  |
|  | 41-45 | 1.48228 | 0.70859 |  |
|  | 46-50 | 1.47956 | 0.81076 |  |
|  | 51-80 | 1.92305 | 0.73188 |  |
| Forest proximity of village | Outside forest | Reference | | <0.001 |
|  | Forest fringe | 0.77026 | 0.39881 |  |
|  | Inside forest | 2.59209 | 0.36768 |  |
| Material of roof | Grass/leaves | -0.03273 | 0.67613 | <0.01 |
|  | Tent | 1.35235 | 0.48477 |  |
|  | Corrugated iron | Reference | |  |
|  | Wood planks/ cement/tiles | -0.70368 | 0.26758 |  |
| Household owns a toilet | No | Reference |  | 0.06 |
|  | Yes | -0.35946 | 0.18914 |  |
| Household owns buffaloes | No | Reference | | <0.01 |
|  | Yes | 0.82853 | 0.28977 |  |
| Household head had received information on malaria via TV in the past 3 months | No | Reference | | <0.01 |
|  | Yes | -1.02219 | 0.40875 |  |
| Insecticides had been sprayed inside the house in the past year | No | Reference | | <0.01 |
|  | Yes | -0.47608 | 0.18622 |  |
| Having slept outside last night | No | Reference | | 0.07 |
|  | Yes | 0.72020 | 0.39234 |  |
| Having slept under a net last night | No | Reference |  | 0.94 |
|  | Yes | -0.01898 | 0.24718 |  |
| Work-unrelated travels overnight in the last month to… | None | Reference | | <0.05 |
|  | Field sites | -0.52234 | 0.48253 |  |
|  | Forest sites | 0.68471 | 0.22425 |  |
|  | A village | 0.14994 | 0.27518 |  |
|  | A city | -1.42758 | 1.06795 |  |
|  | Unspecified | 0.12539 | 0.41792 |  |
| Work in the last two months… |  |  |  |  |
| …in the deep forest | No | Reference | | <0.001 |
|  | Yes | 0.95113 | 0.27823 |  |
| …in a cassava field | No | Reference |  | <0.05 |
|  | Yes | 0.31620 | 0.15641 |  |

**Table ST4** Details of spatial clusters identified by covariate-adjusted SaTScan analysis.

| **Cluster** | **Lat** | **Long** | **Radius** | **# Pop** | **# Obs** | **# Exp** | **RR** | ***p*** |
| --- | --- | --- | --- | --- | --- | --- | --- | --- |
| 1 | 12.22155 | 106.9107 | 4.5 km | 273 | 80 | 37 | 2.5 | <0.001 |
| 2 | 12.24500 | 106.8488 | 2.5 km | 152 | 60 | 28 | 2.4 | <0.001 |

**Table ST5** Portion of villages that form the spatial clusters in the SaTScan analysis.

| **Village** | **Cluster** | **# households** | **# households in cluster** | **% of households in cluster** |
| --- | --- | --- | --- | --- |
| Beng-Gaty | 1 | 42 | 37 | 88.1% |
| Sraelvy | 1 | 36 | 20 | 55.6% |
| Ohtrone | 1 | 23 | 19 | 82.6% |
| Ohchra | 2 | 39 | 39 | 100% |

**Supplementary Figures**


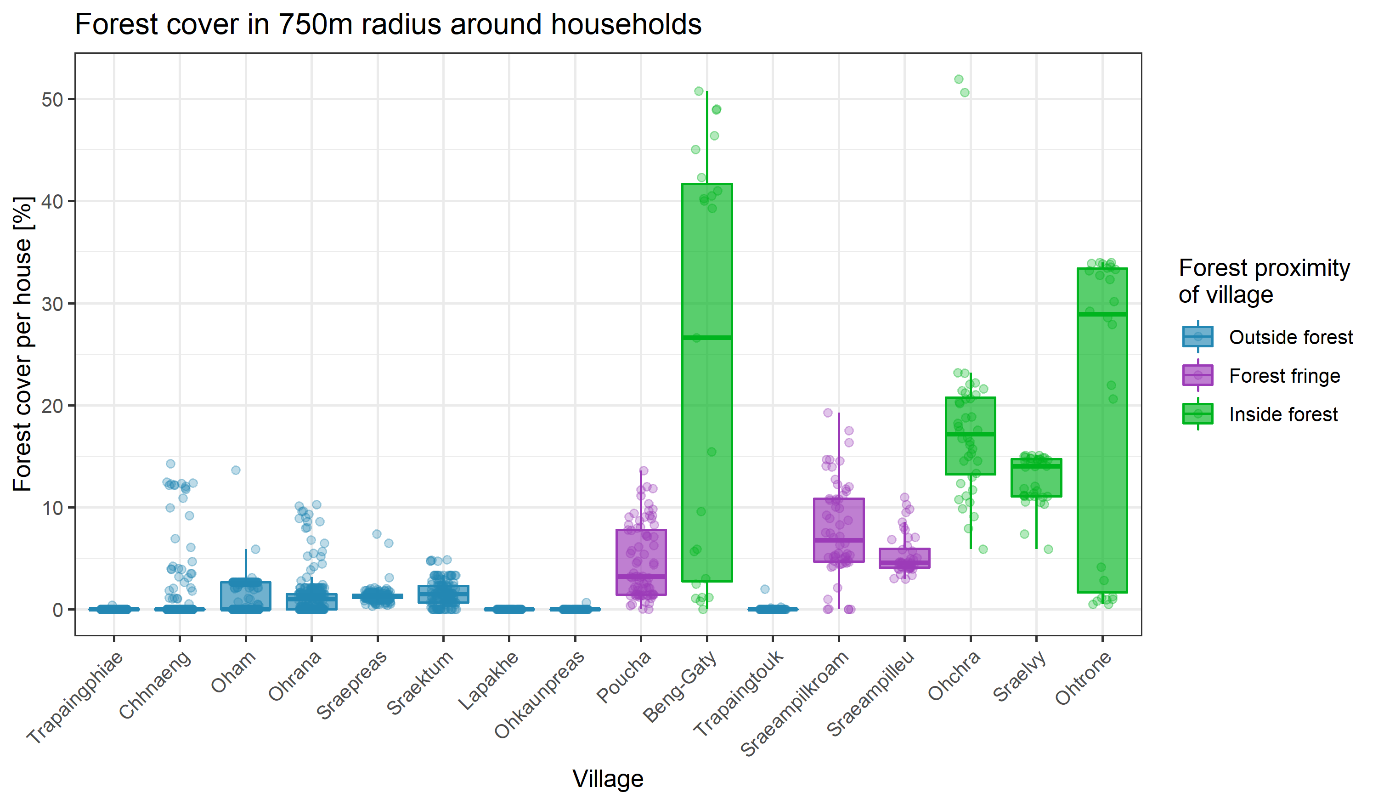


**Figure S1** Classification of villages as to their forest proximity based on forest cover in 750 m vicinity around census households. Forest cover was calculated by remote sensing from satellite imagery as in [1].


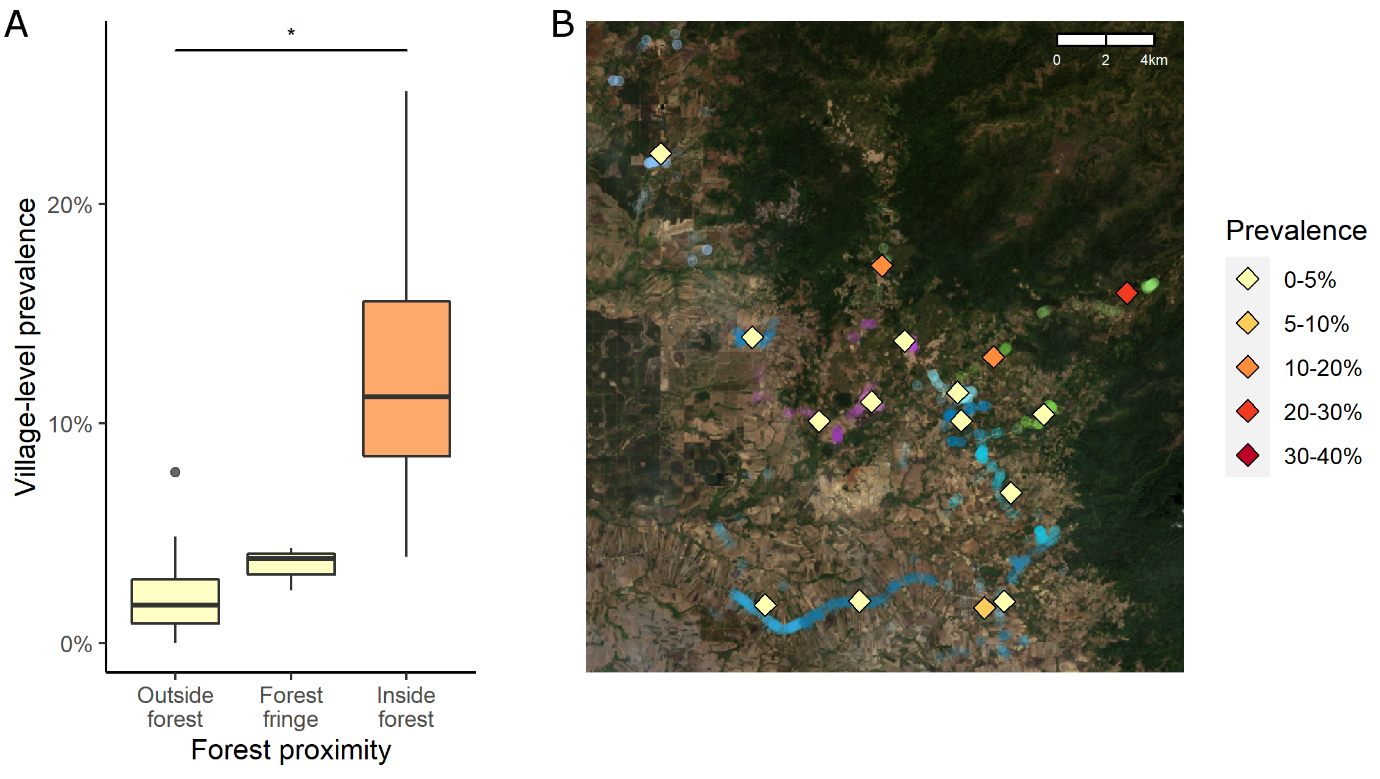


**Figure S2** Prevalence of P. falciparum infection per village (as boxplots by proximity of villages to the forest in panel A and as squares in panel B). Household locations of survey participants in the map background, transparently coloured by village in shades of blue, purple, or green if village is in category “outside forest”, “forest fringe”, or “inside forest”, resp. Significance asterisk for Kruskal-Wallis test of differences in village-level prevalence.


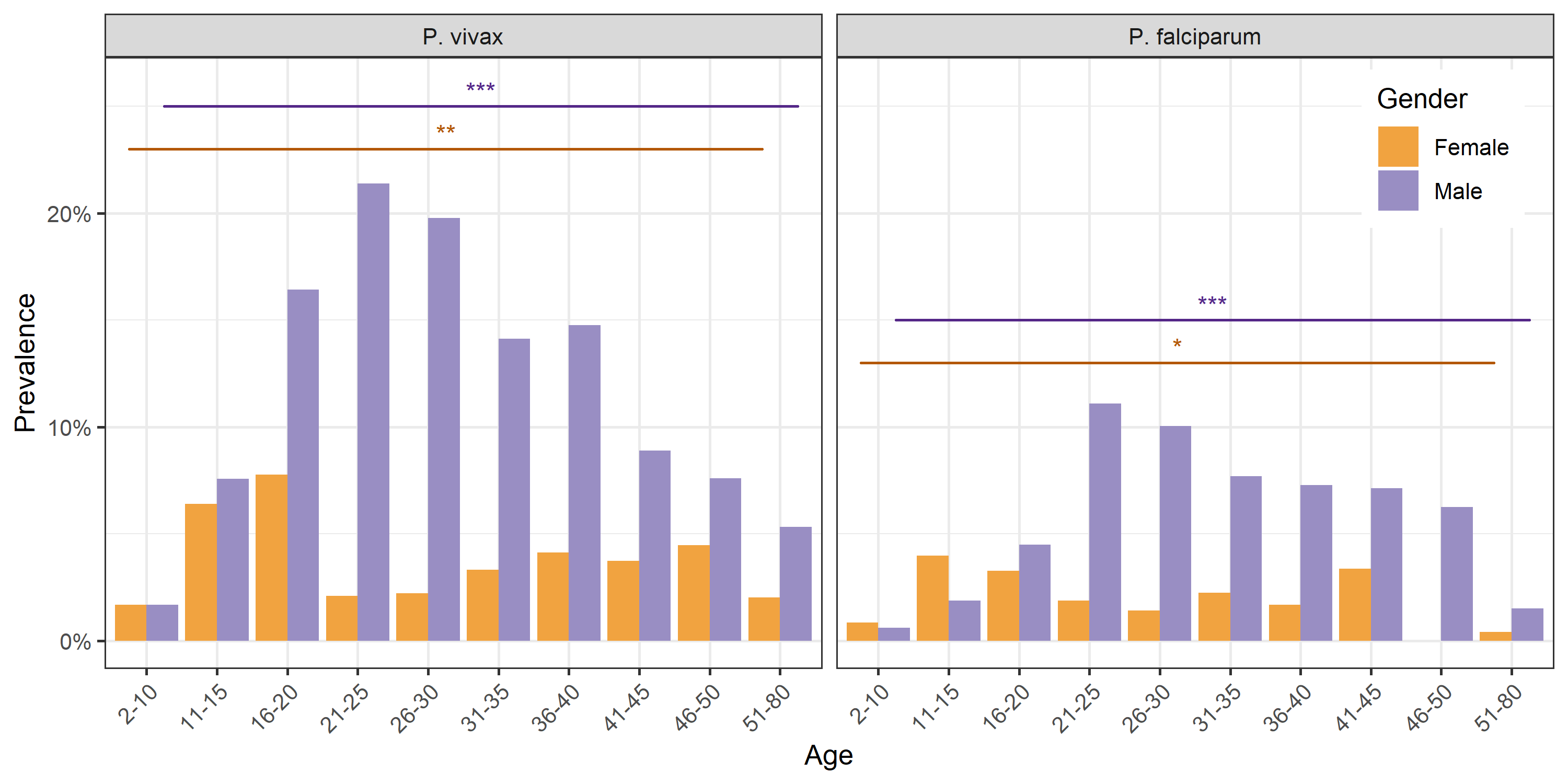


**Figure S3** Prevalence of infection by age per gender and species. Significance asterisks for test of differences in prevalence across age groups per gender strata.

**References**

1. Pepey A, Souris M, Vantaux A, Morand S, Lek D, Mueller I, et al. Studying Land Cover Changes in a Malaria-Endemic Cambodian District: Considerations and Constraints. Remote Sens. 2020;12:2972.
